# Comparative performances of the Qvella FAST system and conventional methods for rapid identification and antibiotic susceptibility testing on monomicrobial positive blood cultures

**DOI:** 10.1128/jcm.01332-24

**Published:** 2024-12-20

**Authors:** Malo Penven, Manon Louazon, Charlotte Freret, Alexandra Sauron, Meghane Pilard, Elisa Creignou, Ophélie Gardan, Maryne Haumont, Asma Zouari, Stéphane Lorre, Vincent Cattoir

**Affiliations:** 1 Department of Bacteriology, Rennes University Hospital 36684 , Rennes, France; 2 UMR_S1230 Inserm BRM, University of Rennes 27079 , Rennes, France; 3 National Reference Center for Antimicrobial Resistance (lab ‘Enterococci’), Rennes University Hospital 36684 , Rennes, France; NorthShore University HealthSystem Department of Pathology and Laboratory Medicine, Evanston, Illinois, USA

**Keywords:** bloodstream infection, BSI, sepsis, bacteremia, BC, rapid diagnosis, ID, AST

## Abstract

Rapid and accurate diagnosis of sepsis is of paramount importance to reduce associated morbidity and mortality. The Qvella FAST System is a new instrument that concentrates and purifies bacteria from positive-flagged blood culture bottles (PFBCBs) to produce a “liquid” colony comparable to a subcultured colony in less than 40 min for rapid ID and calibrated antibiotic susceptibility testing (AST). In this study, we evaluated performances of the FAST System workflow and our rapid routine manual workflow (bacterial pellet obtained after lysis, cleaning, washing, and centrifugation for ID; AST by disc diffusion by direct inoculation after dilution) by comparison to the reference method based on 24-h bacterial subcultures. Two panels of PFBCBs were studied: panel A (including 107 prospective BCs from septic patients, October–November 2022) and panel B (including 102 BCs spiked with difficult-to-identify bacteria [mostly streptococci] and multidrug-resistant isolates), resulting in a total of 209 evaluable samples. The FAST System provided a correct ID to the species level in 178/209 (85.2%) of cases. For AST, the categorical agreement (CA) of the FAST System was 99.4%, with rates of very major (VME), major (ME), and minor (mE) errors of 0.59%, 0.20%, and 0.26%, respectively. Our rapid routine workflow based on manual methods show similar results for ID (86.2%) and AST (CA, 99.6%; VME, 0.50%; ME, 0.16%; mE, 0.13%). In conclusion, the Qvella FAST system, a promising tool that can reduce diagnostic time by approximately 1 day, shows excellent performances for rapid ID and AST.

## INTRODUCTION

Bacteremia is defined by the presence of viable bacteria in the bloodstream, which can trigger a dysregulated systemic inflammatory and immune response, commonly known as sepsis (1). Every year, 49 million people suffer from sepsis associated with 11 million sepsis-related deaths worldwide, accounting for one-fifth of all death (2). This leading cause of mortality is associated with significant healthcare expenditure, estimated to be $38 billion annually in the United States (3). The cost of sepsis per patient ranges between $16,324 and $38,298 and is directly correlated to its severity: from sepsis to septic shock (4). Antibiotic treatment is the cornerstone of sepsis management, and there is a relationship between the timing of antibiotic initiation and patient outcomes. Thus, for every hour without appropriate antibiotic administration, the survival rate decreases by approximately 7% (5). Consequently, fast and accurate identification (ID) and antibiotic susceptibility testing (AST) of the bacterial etiological agent are therefore of paramount importance. Rapid AST is increasingly important considering the rise of multi-drug-resistant (MDR) bacteria, which are associated with empirical antibiotic treatment failure (6). Traditionally, the management of positive-flagged blood culture bottles (PFBCBs) is based on Gram staining, ID through a 24-h bacterial subculture, and a minimum of 48 h for AST results. For decades, clinical microbiology laboratories have strived to reduce the turnaround time for PFBCB processing in order to decrease patient mortality and healthcare facility costs. Thus, various manual methods have been developed to concentrate and purify bacterial cells from PFBCBs, facilitating ID (currently performed by MALDI-TOF mass spectrometry in many laboratories) and AST. This rapid workflow provides a time saving of 24 h compared with overnight subculture-based routine workflows (7–11). Over the past decade, technological advances had led to the development of new approaches based on multiplex PCR, microscopic imaging analysis fluorescence hybridization *in situ*, flow cytometry or detection of volatile organic compounds released during bacterial growth (12–17). More recently, the FAST System (Qvella) has been developed to provide a liquid colony (LC) in less than 40 min from PFBCBs, comparable to a 24-h subculture. The LC allows purification of bacterial cells for ID and calibrated AST (0.5 McFarland), similarly to what is obtained from colonies isolated on a solid medium.

The aim of this study was to compare performances for rapid ID and AST of the Qvella FAST system and a manual rapid method routinely used in the laboratory by using the subculture-based method as reference.

## MATERIALS AND METHODS

### Samples and study design

**Fig 1:**
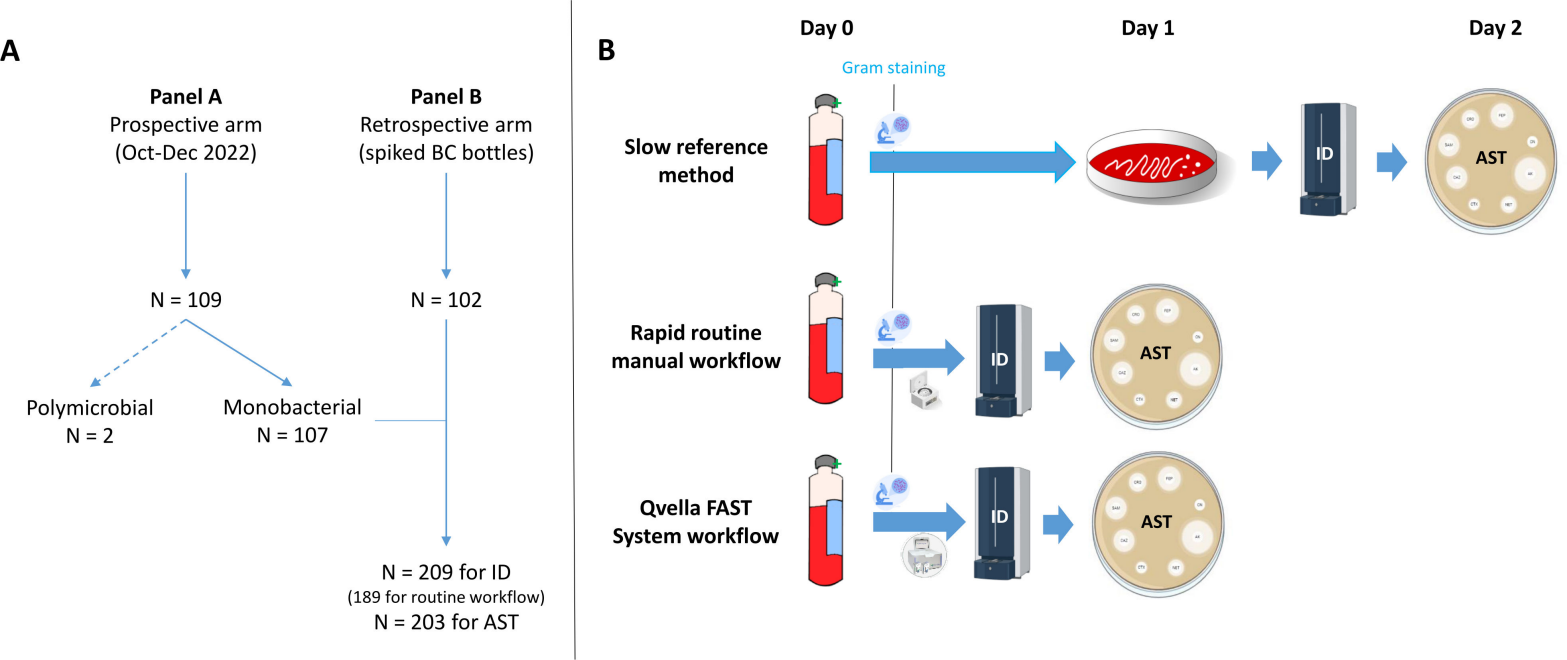
(A) Flowchart of the study. (B) Different approaches used in the study.

This study was carried out by using two different panels of PFBCBs. The panel A (*n* = 109) corresponded to blood cultures (BD BACTEC blood culture bottles: Plus aerobic, Plus anaerobic, Peds Plus; Becton Dickinson, Heidelberg, Germany) prospectively collected from septic patients across various wards of the Rennes University Hospital from October 2022 to December 2022 (Fig. 1A). These blood samples were collected following national recommendations for bacteremia diagnosis. Note that the following exclusion criteria were applied: samples from patients that had already been included in the study and postmortem BCs. The panel B (*n* = 102) was composed of BC bottles (BD BACTEC Plus Aerobic) that were spiked with difficult-to-identify bacterial species (*i.e.*, *Stenotrophomonas maltophilia* and streptococci) or MDR isolates (Fig. 1A; Table 1). Characterization of resistance mechanisms had been performed phenotypically by disk diffusion (antibiogram, synergy tests for ESBL, and AmpC hyperproduction) or immunochromatographic tests (detection of PLP2a [Clearview PLP2a; Abbot, Green oaks, USA], CTX-M [CTX-M multi; NG Biotech, Woden, Australia], and carbapenemases [CARBA-5; NG Biotech, Woden, Australia]) and genotypically by PCR (detection of *vanA* and *vanB*). The spiking protocol employed an inoculation of 10 mL of human blood collected from healthy volunteers with a standardized inoculum of approximately 125 bacterial colony-forming units (CFUs). This study was carried out during the opening hours of the laboratory (8 am to 8 pm). Note that the median time from bottle flagged positive to processing was 3 h 53 (00 h 02–14 h 17) with only 35 (16.7%) bottles processed after a <2-h positivity.

**TABLE 1:** Strains used for spiked blood culture bottles (panel B)

Gram-negative bacteria (*n* = 52)	No.
Carbapenem-resistant *Acinetobacter baumannii*	4
ESBL^*a*^-producing *Enterobacterales*	10
*Citrobacter freundii*	2
*Citrobacter koseri*	1
*Enterobacter cloacae* complex	1
*Escherichia coli*	3
*Klebsiella pneumoniae*	3
AmpC-hyperproducing *Enterobacterales*	8
*Citrobacter freundii*	2
*Enterobacter cloacae* complex	3
*Hafnia alvei*	1
*Morganella morganii*	2
Carbapenemase-producing *Enterobacterales*	20
*Citrobacter freundii* (1 VIM, 2 OXA-48-like)	3
*Enterobacter cloacae* complex (1 VIM, 3 NDM, 2 OXA-48-like)	5
*Escherichia coli* (3 NDM, 4 OXA-48-like)	7
*Klebsiella pneumoniae* (1 NDM, 4 OXA-48-like)	5
Difficult-to-treat *Pseudomonas aeruginosa*^*b*^	5
*Stenotrophomonas maltophilia*	5

Gram-positive bacteria (*n* = 50)	No.
Vancomycin-resistant enterococci	10
*Enterococcus faecalis* (VanA)	2
*Enterococcus faecium* (3 VanA, 2 VanB)	5
*Enterococcus raffinosus* (VanA)	1
*Enterococcus durans* (VanA)	1
*Enterococcus avium* (VanA)	1
Methicillin-resistant *Staphylococcus aureus*	10
Alpha-hemolytic streptococci	20
*Streptococcus anginosus* group	5
*Streptococcus mitis*/*oralis*	3
*Streptococcus sanguinis*	2
*Streptococcus pneumoniae*	10
Beta-hemolytic streptococci	10
*Streptococcus agalactiae*	3
*Streptococcus dysgalactiae*	3
*Streptococcus pyogenes*	4

^*a*^ ESBL, extended-spectrum β-lactamase.

^*b*^ *P. aeruginosa* isolate not susceptible to piperacillin–tazobactam, meropenem, imipenem, ceftazidime, cefepime, aztreonam, ciprofloxacin, and levofloxacin.

### Slow reference method

The reference method consisted of an overnight subculture from PFBCBs on Columbia agar supplemented with 5% sheep blood (Oxoid; Thermo Fisher Diagnostics, Daridlly, France) (Fig. 1B). ID was performed from isolated colonies by MALDI-TOF mass spectrometry (see below). AST was carried out in accordance with 2023 “Comité de l’antibiogramme de la Société Française de Microbiologie” (CA-SFM)/EUCAST recommendations (18). Briefly, a bacterial inoculum standardized to 0.5 McFarland was used to perform an antibiogram by disc diffusion on Mueller–Hinton (MH) agar or MH supplemented with 5% defibrinated horse blood and 20 mg/L β-NAD (MHF), depending on bacterial species.

### Rapid routine manual workflow

The rapid routine workflow consisted of manual methods directly performed on PFBCBs (Fig. 1B). For ID, 2 mL of blood culture was lysed with 0.1% Triton followed by vortexing for 30 s. The suspension was then cleaned and washed 2–3 times before being concentrated by centrifugation to obtain a bacterial pellet. The pellet was used for identification by MALDI-TOF mass spectrometry (see below).

For AST, antibiograms were performed by disc diffusion on MH or MHF agar plates using a non-standardized inoculum obtained by simple dilution of PFBCBs (1/50 for Gram-negative bacteria and staphylococci; and 1/5 for streptococci and enterococci), as recommended by the CA-SFM (18).

### Qvella FAST system workflow

The FAST System workflow comprised isolation and concentration of bacteria cells from 2 mL of PFBCBs by using FAST PBS Prep cartridges (Qvella, Richmond Hill, Canada) (Fig. 1B). The cartridge produces a standardized amount of bacteria known as liquid colony (LC) in around 30 min. The volume of LC obtained ranges from 50 to 200 µL, which was used within the hour for ID by MALDI-TOF mass spectrometry (see below) and calibrated AST (0.5 McFarland) by disc diffusion on Mueller–Hinton (MH) agar or MH supplemented with 5% defibrinated horse blood and 20 mg/L β-NAD (MHF) depending on bacterial species.

### Bacterial ID

Bacterial ID was performed by MALDI-TOF mass spectrometry (Sirius; Bruker Daltoniks, Bremen, Germany) from isolated colonies for the reference method, from pellets for the rapid routine workflow and from LCs for the Qvella FAST System workflow. Three spots were performed for LCs and bacterial pellets to optimize bacterial ID, while a single spot was run for the reference method. To improve identification performance, 1.2 µL of formic acid was used prior to the addition of 1.2 µL of HCCA-dispersed IVD Matrix (Bruker Daltoniks, Bremen, Germany). The MALDI-TOF library was MBT compass IVD 4.2 (version 100). The highest scores obtained were considered for comparison. A threshold score of ≥1.8 was selected to ensure accurate ID at the species level.

### AST and comparison assessment

AST was performed by disc diffusion (see above) for all workflows using isolated colonies, diluted BCs, or LCs. Both rapid routine and Qvella FAST System workflows were compared to the reference method. Interpretation was performed using zone diameter breakpoints and clinical categories established by 2023 CA-SFM/EUCAST guidelines (18). Inhibition diameters were compared, and concordance agreement (CA) rates were calculated. Discrepancies were also analyzed, considering the following errors: very major error (VME), major error (ME), and minor error (mE). VME occurred when a strain was falsely categorized as susceptible when the strain was actually resistant. ME arose when a susceptible strain was falsely categorized as resistant. mE corresponded to discrepancies between categories that were more closely related (S⇔I ; I⇔R). The percentage of VMEs was calculated as follows: $\%VME=\frac{numberofVME}{totalofresistantisolates}\times 100$. The rate of MEs was computed as follows: $\%ME=\frac{numberofME}{totalofsusceptibleisolates}\times 100$. To calculate the rate of mEs, the total number of mEs was divided by the total number of antibiotics tested ×100. Statistical analysis was performed using GrapPad Prism v.5 (GraphPad Software Inc., San Diego, CA, USA). Fisher exact test was used to compare the different parameters, and *P* < 0.05 was considered as significant.

## RESULTS

### Study samples

For panel A, 109 PFBCBs were prospectively analyzed, including two polymicrobial cultures that were excluded. Among the 107 remaining samples, a total of 58 (54.2%) Gram-negative bacteria were isolated, with the majority belonging to the order *Enterobacterales* (*n* = 52, 48.6%), with a large predominance of *Escherichia coli* (*n* = 36, 33.6%) (Table 2). Among Gram-positive bacteria (*n* = 49, 45.8%), 15 (14%) isolates of *Staphylococcus aureus* and 24 (22.4%) non-*aureus* staphylococci grew (Table 2). A small number of enterococci (*n* = 4) and streptococci (*n* = 4) were also identified (Table 2). Altogether, with the 102 PFBCBs of the panel B (Table 1), a total of 209 samples were included for downstream analysis (including three failed initial runs that were resolved after reprocessing).

**TABLE 2:** Bacterial species identified by the reference method from PFBCBs prospectively collected from septic patients (panel A)

Gram-negative bacteria (*n* = 58)	No.
*Enterobacterales*	52
*Escherichia coli*	36
*Citrobacter koseri*	1
*Enterobacter cloacae* complex	4
*Proteus mirabilis*	3
*Klebsiella pneumoniae*	6
*Salmonella* sp.	1
Non-fermenting	7
*Pseudomonas aeruginosa*	6
*Achromobacter xylosoxidans*	1

Gram-positive bacteria (*n* = 49)	No.
Enterococci	4
*Enterococcus faecalis*	1
*Enterococcus faecium*	2
*Enterococcus hirae*	1
*Staphylococcus aureus*	15
Non-*aureus* staphylococci	24
*Staphylococcus epidermidis*	15
*Staphylococcus haemolyticus*	3
*Staphylococcus hominis*	3
*Staphylococcus capitis*	1
*Staphylococcus petrasii*	1
*Staphylococcus warneri*	1
Streptococci	4
*Streptococcus anginosus* group	1
*Streptococcus mitis*/*oralis*	1
*Streptococcus vestibularis*	1
*Streptococcus pneumoniae*	1
Gram-positive rods	2
*Clostridium perfringens*	1
*Corynebacterium striatum*	1

### ID comparison

Performances of ID by MALDI-TOF were evaluated on 209 samples for the FAST System but only on 189 samples for the routine manual method due to organizational reasons (unavailability of trained staff). The overall concordance for ID with the reference technique (culture-grown colonies) was very good in both techniques. Thus, species-level ID was produced from pellet and the LC in 86.2% (163/189) and 85.2% (178/209) of cases, respectively (*P* = 0.78) (Table 3). The delay between positivity and processing of the PFBCBs influenced ID performances, resulting in lower results in the first 2 h (Table 4). Indeed, correct IDs were obtained in only 73.3% (22/30) and 71.4% (25/35) of cases for the manual method and LC, respectively (with no difference between the two approaches), while ID performances were >85% when the time was >2 h (Table 4).

**TABLE 3:** Performances of MALDI-TOF identification using the routine method versus the liquid colony obtained by the FAST System^*a*^

Bacterial species	Manual method	Liquid colony	P
Gram-positive bacteria	68/89 (76.4%)	78/99 (78.8%)	0.73
Enterococci	11/14 (78.6%)	13/14 (92.9%)	0.60
*Enterococcus avium*	0/1 (0%)	1/1 (100%)	
*Enterococcus durans*	1/1 (100%)	1/1 (100%)	
*Enterococcus faecalis*	3/3 (100%)	3/3 (100%)	
*Enterococcus faecium*	6/7 (85.7%)	6/7 (85.7%)	
*Enterococcus hirae*	1/1 (100%)	1/1 (100%)	
*Enterococcus raffinosus*	0/1 (0%)	1/1 (100%)	
Staphylococci	37/41 (90.24%)	45/49 (91.8%)	1.00
*Staphylococcus aureus*	23/25 (92.0%)	24/25 (96.0%)	
*Staphylococcus capitis*	1/1 (100%)	1/1 (100%)	
*Staphylococcus epidermidis*	8/9 (88.9%)	12/15 (80.0%)	
*Staphylococcus haemolyticus*	1/2 (50.0%)	3/3 (100%)	
*Staphylococcus hominis*	3/3 (100%)	3/3 (100%)	
*Staphylococcus petrasii*	1/1 (100%)	1/1 (100%)	
*Staphylococcus warneri*	nd	1/1 (100%)	
Streptococci	18/32 (56.3%)	18/34 (52.9%)	0.81
*Streptococcus mitis/oralis*	3/3 (100%)	2/3 (66.7%)	
*Streptoccocus agalactiae*	3/3 (100%)	3/3 (100%)	
*Streptococcus anginosus* group	2/5 (40.0%)	3/6 (40.0%)	
*Streptococcus dysgalactiae*	2/3 (66.7%)	2/3 (66.7%)	
*Streptococcus pneumoniae*	3/10 (30.0%)	2/11 (18.2%)	
*Streptococcus pyogenes*	3/4 (75.0%)	3/4 (75.0%)	
*Streptococcus salivarius*	0/2 (0%)	1/2 (50%)	
*Streptococcus vestibularis*	2/2 (100%)	2/2 (100%)	
Other Gram-positive bacteria	2/2 (100%)	2/2 (100%)	
*Clostridium perfringens*	1/1 (100%)	1/1 (100%)	
*Corynebacterium striatum*	1/1 (100%)	1/1 (100%)	
Gram-negative bacteria	95/100 (95%)	100/110 (90.9%)	1.00
Enterobacterales	77/80 (96.25%)	85/89 (95.5%)	
*Citrobacter freundii*	7/7 (100%)	7/7 (100%)	
*Citrobacter koseri*	1/2 (50.0%)	2/2 (100%)	
*Enterobacter cloacae*	9/11 (81.8%)	11/12 (91.7%)	
*Enterobacter asburiae*	1/1 (100%)	1/1 (100%)	
*Escherichia coli*	41/41 (100%)	44/46 (95.7%)	
*Hafnia alvei*	1/1 (100%)	1/1 (100%)	
*Klebsiella pneumoniae*	12/12 (100%)	13/14 (92.8%)	
*Morganella morganii*	2/2 (100%)	2/2 (200%)	
*Proteus mirabilis*	2/2 (100%)	3/3 (100%)	
*Salmonella sp*	1/1 (100%)	1/1 (100%)	
Non-fermenting	18/20 (90%)	15/21 (71.4%)	0.24
*Achromobacter xylosoxidans*	nd	1/1 (100%)	
*Acinetobacter baumannii*	4/4 (100%)	4/4 (100%)	
*Pseudomonas aeruginosa*	10/11 (90.9%)	9/11 (81.8%)	
*Stenotrophomonas maltophilia*	4/5 (80.0%)	1/5 (20.0%)	
Total	163/189 (86.2%)	178/209 (85.2%)	0.78

^*a*^ nd, not determined.

**TABLE 4:** Performances of MALDI-TOF identification according to the time between bottle positivity and processing

Time between bottle positivity and processing	*N*	ID (*n* [%])		*P*
		Manual method	LC	
≤2 h	35	22/30 (73.3%)	25/35 (71.4%)	1
>2–≤6 h	69	54/61 (85.5%)	59/69 (85.5%)	0.79
>6–≤10 h	57	47/53 (88.6%)	52/57 (91.2%)	0.76
>10–≤16 h	48	40/45 (88.9%)	42/48 (87.5%)	1

Three spots were performed to optimize the chance of ID using rapid methods. Correct ID was obtained with 3/3 spots in 64% (121/189) and 74.2% (155/209), 2/3 in 14.8% (28/189) and 6.22% (13/209), and 1/3 in 7.4% (14/189) and 3.8% (8/209) of cases using the pellet and the LC, respectively. No ID discrepancies were found between the three spots in any case.

All ID failures with the FAST System (*n* = 31) or the manual method (*n* = 26) were attributed to the absence of peaks or an ID score lower than 1.8. ID performances of both approaches seemed to be higher for Gram-negative bacteria (>90%) than for Gram-positive bacteria (<79%) (Table 3). Indeed, streptococci were the greatest challenge with only 56.3% (18/32) and 52.9% (18/34) of correct ID by manual method and LCs, respectively (*P* = 0.81) (Table 3). Specifically, *S. pneumoniae* exhibited poor performance, with a concordant identification in only 3/10 (30.0%) isolates with manual method and 2/11 (18.2%) isolates using the FAST System (Table 3). Note that ID performance for *S. maltophilia* was also lower from the LC (1/5, 20%) than the manual method (4/5, 80%) (Table 3). Hence, the majority of ID failures were related to streptococci for routine and FAST System workflows, respectively, representing 53.8% (14/26) and 51.6% (16/31) of cases. Interestingly, no discordant ID results were obtained in this study, neither for the manual method nor for the FAST Sytem. When the ID was correctly obtained, the mean MALDI-TOF score was 2.16 (SD ± 0.18) for the manual method and 2.18 (SD ± 0.18) for the FAST system. These results were slightly lower, with an average score of 2.26 (SD ± 0.17), compared with those obtained on subcultured colonies.

### AST comparison

For the AST comparison, six samples were excluded: an isolate of *Clostridium perfringens* (absence of CA-SFM/EUCAST recommendations for anaerobes by simple dilution), two isolates of streptococci (*Streptococcus oralis* and *S. pneumoniae*) with no adequate inoculum (<0.5 McFarland), and three technical errors (*i.e.*, contamination).

The comparative analysis of AST was performed on a highly diverse collection of bacterial species with a wide range of antibiotics, enabling 3,899 antibiotic/bacteria combinations for each technique. Globally, the CA was very high for both techniques: 99.6% (3884/3899) for the manual method and 99.4% (3877/3899) for the FAST System (*P* = 0.3) (Table 5). Similarly, CA was excellent in both prospective and retrospective arms (panels A and B), with a bacterial population enriched with MDR bacteria and streptococci (CA for panel B > 99%) (Table 5). The results were equally convincing for both Gram-positive and Gram-negative bacteria (CA > 99%) (Table 5). The rates of VME and ME were very low: 0.50% (6/1175) and 0.16% (4/2415) with the manual method, and 0.59% (7/1176) and 0.20% (5/2450) for the FAST System (Table 5). Moreover, only a few mEs were noted, representing 0.13% (5/3899) and 0.26% (10/3899) for manual and FAST System workflows, respectively (Table 5). For all these parameters, no significant differences were observed between the two approaches for all the subgroups selected (Table 5). The analysis of discrepancies showed that errors were not associated to a specific class of antibiotics but gathered across β-lactams, macrolides, tetracyclines, quinolones, and furans (Table 6). It should be noted that the majority of discrepancies were consistent across both methods, suggesting that errors may be related to the isolate.

**TABLE 5:** Categorical agreements (CAs) of drug–bacteria combinations between routine or FAST Sytem versus reference method

Group	AST agreement	Routine/reference		LC/reference		*P*
		*N*	%	*N*	%	
Panel A	CA	2,217	99.73	2,215	99.64	0.79
	VME	4	1.01	3	0.76	1
	ME	1	0.06	1	0.06	1
	mE	1	0.04	4	0.18	0.37
Panel B	CA	1,667	99.46	1,662	99.16	0.4
	VME	2	0.26	4	0.51	0.69
	ME	3	0.37	4	0.5	1
	mE	4	0.24	6	0.36	0.75
Gram +	CA	1,188	99.4	1,185	99.16	0.63
	VME	4	1.08	5	1.35	1
	ME	2	0.26	2	0.26	1
	mE	1	0.08	3	0.25	0.75
Gram -	CA	2,696	99.7	2,692	99.56	0.5
	VME	2	0.25	2	0.25	1
	ME	2	0.12	3	0.18	1
	mE	4	0.15	7	0.26	0.55
Total	CA	3,884	99.62	3,877	99.44	0.32
	VME	6	0.51	7	0.59	1
	ME	4	0.16	5	0.2	1
	mE	5	0.13	10	0.26	0.3

**TABLE 6:** Drug–bacteria combinations with significant discrepancies

Very major errors			
Manual method (0.50%)		Liquid colony (0.59%)	
Antibiotic	Species	Antibiotic	Species
Erythromycin	*S. epidermidis^a^*	Erythromycin	*S. epidermidis^a^*
Clindamycin	*S. epidermidis^a^*	Clindamycine	*S. epidermidis^a^*
Nitrofurantoin	*E. faecium^a^*	Nitrofurantoin	*E. faecium^a^*
Tetracycline	*S. epidermidis*	Tetracycline	*S. agalactiae*
Tobramycin	*P. aeruginosa^a^*	Penicillin	*S. oralis*
Ertapenem	*C. freundii^a^*	Tobramycin	*P. aeruginosa^a^*
		Ertapenem	*C. freundii^a^*

Major errors			
Manual method (0.16%)		Liquid colony (0.20%)	
Antibiotic	Species	Antibiotic	Species
Mecillinam	*E. coli* ^*a*^	Mécillinam	*E. coli^a^*
Norfloxacin	*E. faecium* ^*a*^	Norfloxacin	*E. faecium^a^*
Trimethoprim–sulfamethoxazole	*E. faecium* ^*a*^	Trimethoprim–sulfamethoxazole	*E. faecium^a^*
Fosfomycin	*K. pneumoniae*	Mecillinam	*K. pneumoniae*
		Fosfomycin	*K. pneumoniae*

^*a*^ Bacterial isolates involved in VME/ME observed in both techniques.

## DISCUSSION

Decreasing the time for sepsis diagnosis is paramount for reducing its impact on mortality and healthcare costs. The aim of this study was to evaluate the performance of the FAST System to accelerate the monitoring of PFBCBs for rapid and accurate bacterial identification and AST. In this work, we conducted a head-to-head comparison of the FAST System with the reference method based on a 24-h subculture. In parallel, we also performed a comparison with our rapid workflow based on manual methods. Overall, the FAST system demonstrated very good performance for rapid ID, achieving a species-level identification in 85.2% of cases. However, these results are lower than those reported in the literature, with correct ID rates varying between 87.7% and 100% (19–25). The lower performance observed here may be attributed to the original study design, which included an arm (panel B) enriched with bacterial species that are challenging to identify, such as streptococci. Indeed, in the prospective arm, species-level identification was achieved in 91.5% of cases. In total, only 52.9% (18/34) streptococci and 2/11 (18.2%) pneumococci were correctly identified, highlighting the challenge of identifying streptococcal species from PFBCBs. The manual method showed similar results, with 86.2% correct identification (*P* = 0.78). However, poorer results (<75% of correct IDs) were observed if the PFBCBs were processed within the first 2 h after bottle positivity. Both methods provide a time-saving advantage of approximately 24 h compared with the workflow based on bacterial subculture. It should be noted that Sy and colleagues showed better results of the FAST System to ensure bacterial ID than another widely used rapid technique, the SepsiTyper (Bruker Daltoniks, Bremen, Germany), for both Gram-positive (97.4% vs 79.9%) and Gram-negative bacteria (95.7% vs. 91.4%) (20).

In our study, performances of the FAST System to achieve a standardized AST in disc diffusion were excellent compared with the reference method, with a CA rate of 99.16% and 99.56%, a VME rate of 1.35% and 0.18%, an ME rate of 0.26% and 0.18%, and an mE rate of 0.25% and 0.26% for Gram-positive and Gram-negative bacteria, respectively. These results are in line with those published in previous studies, where the FAST system has also demonstrated favorable results for disc diffusion and automated AST systems (Phoenix, Vitek 2, and MicroScan systems) or broth microdilution (Merlin plates), which have reported CA > 96% for both Gram-positive and Gram-negative bacteria, respectively (19–25).

To our knowledge, this is the first study to evaluate the FAST System in comparison to rapid workflow conducted directly on PFBCBs (CASFM/EUCAST), yielding comparable findings.

From a practical point of view, the main difference between these two workflows relies in the centrifugation, lysis, and washing steps, which are not automated in the home-made technique compared with the Qvella FAST System. For the FAST System, the technical time varies between 20 to 40 min for one or two BC bottles with 2 and 4 min of hands-on time, respectively. For the manual workflow, the time varies between 30 and 60 min for up to 12 positive BC bottles. Concerning the cost of the techniques, the manual method is obviously much lower than that of the Qvella FAST System but the latter does not necessitate a technical expertise.

Other aspects of LC can be highlighted, although they were not evaluated in this study. For example, the LC allows rapid detection of bacterial resistance determinants by lateral flow immunoassays, such as carbapenemases or PBP2a (22, 23). In addition, this technique has also shown satisfactory results for the management of yeasts (25).

In summary, the FAST System is a reliable approach to saving time in the management of PFBCBs, comparable to manual methods that have been used for decades. It is easy to use, available 24/7, and can meet standardization requirements. However, its implementation must take into account the additional costs, especially compared with manual techniques.

## SUPPLEMENTAL MATERIAL

Supplemental tables jcm.01332-24-s0001.pdf Tables S1 and S2.

ASM does not own the copyrights to Supplemental Material that may be linked to, or accessed through, an article. The authors have granted ASM a non-exclusive, world-wide license to publish the Supplemental Material files. Please contact the corresponding author directly for reuse.

## References

[1] Hotchkiss RS, Moldawer LL, Opal SM, Reinhart K, Turnbull IR, Vincent J-L. 2016. Sepsis and septic shock. Nat Rev Dis Primers 2:16045. doi:10.1038/nrdp.2016.45 28117397 PMC5538252

[2] Rudd KE, Johnson SC, Agesa KM, Shackelford KA, Tsoi D, Kievlan DR, Colombara DV, Ikuta KS, Kissoon N, Finfer S, Fleischmann-Struzek C, Machado FR, Reinhart KK, Rowan K, Seymour CW, Watson RS, West TE, Marinho F, Hay SI, Lozano R, Lopez AD, Angus DC, Murray CJL, Naghavi M. 2020. Global, regional, and national sepsis incidence and mortality, 1990–2017: analysis for the Global Burden of Disease Study. The Lancet 395:200–211. doi:10.1016/S0140-6736(19)32989-7 PMC6970225 31954465

[3] Hollenbeak CS, Henning DJ, Geeting GK, Ledeboer NA, Faruqi IA, Pierce CG, Thomas CB, O’Neal HR Jr. 2023. Costs and consequences of a novel emergency department sepsis diagnostic test: the IntelliSep Index. Crit Care Explor 5:e0942. doi:10.1097/CCE.0000000000000942 37465702 PMC10351935

[4] Paoli CJ, Reynolds MA, Sinha M, Gitlin M, Crouser E. 2018. Epidemiology and costs of sepsis in the United States—an analysis based on timing of diagnosis and severity level. Crit Care Med 46:1889–1897. doi:10.1097/CCM.0000000000003342 30048332 PMC6250243

[5] Kumar A, Roberts D, Wood KE, Light B, Parrillo JE, Sharma S, Suppes R, Feinstein D, Zanotti S, Taiberg L, Gurka D, Kumar A, Cheang M. 2006. Duration of hypotension before initiation of effective antimicrobial therapy is the critical determinant of survival in human septic shock. Crit Care Med 34:1589–1596. doi:10.1097/01.CCM.0000217961.75225.E9 16625125

[6] Kalın G, Alp E, Chouaikhi A, Roger C. 2023. Antimicrobial multidrug resistance: clinical implications for infection management in critically Ill patients. Microorganisms 11:2575. doi:10.3390/microorganisms11102575 37894233 PMC10609422

[7] Zengin Canalp H, Bayraktar B. 2021. Direct rapid identification from positive blood cultures by MALDI-TOF MS: specific focus on turnaround times. Microbiol Spectr 9:e0110321. doi:10.1128/spectrum.01103-21 34908465 PMC8672911

[8] Lagacé-Wiens PRS, Adam HJ, Karlowsky JA, Nichol KA, Pang PF, Guenther J, Webb AA, Miller C, Alfa MJ. 2012. Identification of blood culture isolates directly from positive blood cultures by use of matrix-assisted laser desorption ionization-time of flight mass spectrometry and a commercial extraction system: analysis of performance, cost, and turnaround time. J Clin Microbiol 50:3324–3328. doi:10.1128/JCM.01479-12 22875888 PMC3457416

[9] Jonasson E, Matuschek E, Kahlmeter G. 2020. The EUCAST rapid disc diffusion method for antimicrobial susceptibility testing directly from positive blood culture bottles. J Antimicrob Chemother 75:968–978. doi:10.1093/jac/dkz548 32016342 PMC7069491

[10] Banerjee R, Humphries R. 2021. Rapid antimicrobial susceptibility testing methods for blood cultures and their clinical impact. Front Med (Lausanne) 8:635831. doi:10.3389/fmed.2021.635831 33777978 PMC7987685

[11] Christner M, Rohde H, Wolters M, Sobottka I, Wegscheider K, Aepfelbacher M. 2010. Rapid identification of bacteria from positive blood culture bottles by use of matrix-assisted laser desorption-ionization time of flight mass spectrometry fingerprinting. J Clin Microbiol 48:1584–1591. doi:10.1128/JCM.01831-09 20237093 PMC2863888

[12] Altun O, Almuhayawi M, Ullberg M, Ozenci V. 2013. Clinical evaluation of the FilmArray blood culture identification panel in identification of bacteria and yeasts from positive blood culture bottles. J Clin Microbiol 51:4130–4136. doi:10.1128/JCM.01835-13 24088863 PMC3838040

[13] Choi J, Jeong HY, Lee GY, Han S, Han S, Jin B, Lim T, Kim S, Kim DY, Kim HC, Kim E-C, Song SH, Kim TS, Kwon S. 2017. Direct, rapid antimicrobial susceptibility test from positive blood cultures based on microscopic imaging analysis. Sci Rep 7:1148. doi:10.1038/s41598-017-01278-2 28442767 PMC5430693

[14] Rosselin M, Prod’hom G, Greub G, Croxatto A. 2022. Performance evaluation of the quantamatrix QMAC-dRAST system for rapid antibiotic susceptibility testing directly from blood cultures. Microorganisms 10:1212. doi:10.3390/microorganisms10061212 35744730 PMC9229829

[15] Pancholi P, Carroll KC, Buchan BW, Chan RC, Dhiman N, Ford B, Granato PA, Harrington AT, Hernandez DR, Humphries RM, Jindra MR, Ledeboer NA, Miller SA, Mochon AB, Morgan MA, Patel R, Schreckenberger PC, Stamper PD, Simner PJ, Tucci NE, Zimmerman C, Wolk DM. 2018. Multicenter evaluation of the accelerate PhenoTest BC kit for rapid identification and phenotypic antimicrobial susceptibility testing using morphokinetic cellular analysis. J Clin Microbiol 56:e01329-17. doi:10.1128/JCM.01329-17 PMC5869823 29305546

[16] Tibbetts R, George S, Burwell R, Rajeev L, Rhodes PA, Singh P, Samuel L. 2022. Performance of the reveal rapid antibiotic susceptibility testing system on Gram-negative blood cultures at a large urban hospital. J Clin Microbiol 60:e0009822. doi:10.1128/jcm.00098-22 35607972 PMC9199398

[17] Silva-Dias A, Pérez-Viso B, Martins-Oliveira I, Gomes R, Rodrigues AG, Cantón R, Pina-Vaz C. 2021. Evaluation of FASTinov ultrarapid flow cytometry antimicrobial susceptibility testing directly from positive blood cultures. J Clin Microbiol 59:e0054421. doi:10.1128/JCM.00544-21 34346718 PMC8451411

[18] Comité de l’Antibiograme de la Société Française de Microbiologie. Société Française de Microbiologie. Available from: https://www.sfm-microbiologie.org/boutique/comite-de-lantibiograme-de-la-sfm-casfm/. Retrieved 7 May 2024.

[19] Grinberg S, Schubert S, Hochauf-Stange K, Dalpke AH, Narvaez Encalada M. 2022. Saving time in blood culture diagnostics: a prospective evaluation of the Qvella FAST-PBC prep application on the FAST system. J Clin Microbiol 60:e0253321. doi:10.1128/jcm.02533-21 35387489 PMC9116178

[20] Sy I, Bühler N, Becker SL, Jung P. 2023. Evaluation of the Qvella FAST system and the FAST-PBC cartridge for rapid species identification and antimicrobial resistance testing directly from positive blood cultures. J Clin Microbiol 61:e0056923. doi:10.1128/jcm.00569-23 37768103 PMC10595056

[21] Kuo P, LeCrone K, Chiu M, Realegeno S, Pride DT. 2022. Analysis of the FAST system for expedited identification and antimicrobial susceptibility testing of bloodborne pathogens. Diagn Microbiol Infect Dis 104:115783. doi:10.1016/j.diagmicrobio.2022.115783 36031475

[22] Bonaiuto C, Baccani I, Chilleri C, Antonelli A, Giani T, Rossolini GM. 2023. Evaluation of the liquid colony produced by the FAST system for shortening the time of bacterial identification and phenotypic antimicrobial susceptibility testing and detection of resistance mechanisms from positive blood cultures. Diagn (Basel) 13:1849. doi:10.3390/diagnostics13111849 PMC10253117 37296699

[23] Verroken A, Hajji C, Bressant F, Couvreur J, Anantharajah A, Rodriguez-Villalobos H. 2022. Performance evaluation of the FAST System and the FAST-PBC Prep cartridges for speeded-up positive blood culture testing. Front Microbiol 13:982650. doi:10.3389/fmicb.2022.982650 36187982 PMC9520742

[24] Ugaban K, Pak P, She RC. 2022. Direct MALDI-TOF MS and antimicrobial susceptibility testing of positive blood cultures using the FAST system and FAST-PBC Prep cartridges-performance evaluation in a clinical microbiology laboratory serving high-risk patients. Microorganisms 10:2076. doi:10.3390/microorganisms10102076 36296352 PMC9612302

[25] Maddalena C, Giuseppe M, Perez M, Guido S, Stefania S. 2023. Evaluation of the liquid colony for identification and antimicrobial susceptibility testing directly from positive blood cultures. Ann Clin Microbiol Antimicrob 22:72. doi:10.1186/s12941-023-00617-8 37568240 PMC10422792

