# Supplementary material for: Comparative performances of the Qvella FAST system and conventional methods for rapid identification and antibiotic susceptibility testing on monomicrobial positive blood cultures
Source: J Clin Microbiol. 2024 Dec 20;63(2):e01332-24. doi: 10.1128/jcm.01332-24 (PMC11837511; doi:10.1128/jcm.01332-24)
Supplement: Supplemental tables — Tables S1 and S2. [file jcm.01332-24-s0001.pdf]

TABLE S1 Antibiotic susceptibility profiles of Gram-positive bacteria (reference method)

Antibiotic	n	Susceptibility (%)[a]		
		S	I	R
Ampicillin	13	38	8	54
Erythromycin	90	41	2	57
Clindamycin	80	69	1	30
Quinupristin/dalfopristin	57	91	5	04
Linezolid	80	98	0	03
Rifampicin	91	86	1	13
Vamcomycin	44	80	0	20
Teicoplanin	37	95	0	05
Tetracycline	78	79	1	19
Tigecycline	10	100	0	0
Nitrofurantoin	9	67	0	33
Chloramphenicol	13	92	0	08
Norfloxacin	33	79	0	21
Penicillin G	68	34	0	66
Cefoxitin	47	32	0	68
Imipenem	13	0	54	46
Gentamicin	89	73	0	27
Kanamycin	46	48	0	52
Tobramycin	47	47	0	53
Fusidic acid	44	73	0	27
Mupirocin	47	87	0	13
Ciprofloxacin	47	0	47	53
Cotrimoxazole	91	77	0	23
Fosfomycin	1	0	0	100
Oxacillin	7	29	0	71
Levofloxacin	28	32	68	0

[a]S, susceptible, standard dosing regimen; I, susceptible, increased exposure; R, resistant.

TABLE S2 Antibiotic susceptibility profiles of Gram-negative bacteria (reference method)

Antibiotic	n	Susceptibility (%)[a]		
		S	I	R
Ampicillin	88	20	0	80
Amoxicillin-clavulanate	88	50	0	50
Ticarcillin	102	23	5	73
Ticarcillin-clavulanate	128	38	43	19
Piperacillin	161	27	27	46
Piperacillin-tazobactam	20	45	30	25
Mecillinam	84	89	0	11
Cefixime	88	58	0	42
Cefuroxime	51	2	92	6
Cefoxitine	86	65	3	31
Cefotaxime	103	50	1	50
Cefepime	103	58	10	32
Ceftazidime	103	51	8	41
Ceftazidime-avibactam	87	91	0	9
Ertapenem	88	81	0	19
Temocillin	88	0	73	27
Aztreonam	99	58	15	27
Imipenem	103	73	19	08
Meropenem	104	80	12	09
Gentamicin	92	82	0	18
Tobramycin	103	75	0	25
Amikacin	103	91	0	9
Pefloxacin	51	96	0	4
Norfloxacin	88	78	0	22
Ciprofloxacin	103	65	10	25

[a]S, susceptible, standard dosing regimen; I, susceptible, increased exposure; R, resistant.
